# Amylin: Localization, Effects on Cerebral Arteries and on Local Cerebral Blood Flow in the Cat

**DOI:** 10.1100/tsw.2001.23

**Published:** 2001-10-02

**Authors:** Lars Edvinsson, Peter J. Goadsby, Rolf Uddman

**Affiliations:** Department of Internal Medicine, University Hospital, Lund, Sweden

**Keywords:** amylin, adrenomedullin, CGRP, immunocytochemistry, vasomotor responses, cerebral blood flow

## Abstract

Amylin and adrenomedullin are two peptides structurally related to calcitonin gene-related peptide (CGRP). We studied the occurrence of amylin in trigeminal ganglia and cerebral blood vessels of the cat with immunocytochemistry and evaluated the role of amylin and adrenomedullin in the cerebral circulation by in vitro and in vivo pharmacology. Immunocytochemistry revealed that numerous nerve cell bodies in the trigeminal ganglion contained CGRP immunoreactivity (-ir); some of these also expressed amylin-ir but none adrenomedullin-ir. There were numerous nerve fibres surrounding cerebral blood vessels that contained CGRP-ir. Occasional fibres contained amylin-ir while we observed no adrenomedullin-ir in the vessel walls. With RT-PCR and Real-Time-PCR we revealed the presence of mRNA for calcitonin receptor-like receptor (CLRL) and receptor-activity-modifying proteins (RAMPs) in cat cerebral arteries. In vitro studies revealed that amylin, adrenomedullin, and CGRP relaxed ring segments of the cat middle cerebral artery. CGRP and amylin caused concentration-dependent relaxations at low concentrations of PGF2a-precontracted segment (with or without endothelium) whereas only at high concentration did adrenomedullin cause relaxation. CGRP8-37 blocked the CGRP and amylin induced relaxations in a parallel fashion. In vivo studies of amylin, adrenomedullin, and CGRP showed a brisk reproducible increase in local cerebral blood flow as examined using laser Doppler flowmetry applied to the cerebral cortex of the alpha-chloralose-anesthetized cat. The responses to amylin and CGRP were blocked by CGRP8-37. The studies suggest that there is a functional sub-set of amylin-containing trigeminal neurons which probably act via CGRP receptors.

### INTRODUCTION

Amylin is a 37-amino acid peptide that shows 46% amino acid homology with calcitonin gene-related peptide (CGRP) and 15% homology with human calcitonin[6,43]. Since the amylin and CGRP genes are located on chromosome 11 and 12[22,42], respectively, the two genes are probably the result of a duplication of a common ancestral gene. cDNA cloning has revealed that amylin is highly conserved among species[42]. Amylin as well as CGRP exhibit vasodilator activity in peripheral[5] and in cerebral blood vessels[2,10,31]. By immunochemical determination amylin has been reported in the gastrointestinal tract[1,40]. Immunocyto-chemistry has also revealed amylin-ir in neuroendocrine cells in the gastrointestinal tract[37]. In pancreatic islet β-cells, where amylin is expressed in secretory granules, it is co-stored with insulin and co-secreted with insulin in response to stimulation[3,25].

Adrenomedullin is a fairly recently discovered 52-amino acid peptide, originally isolated from human pheochromocytoma, which displays 24% homology with CGRP and shows some homology with amylin[29]. Adrenomedullin relaxes rat and dog cerebral arteries[2,34] via an increase in cAMP[23]. In some tissues adrenomedullin has a vasodilator effect comparable to that of CGRP[2,18.] The CGRP family of peptides has been cloned and contains seven transmembrane domains characteristic for members of the G-protein–linked receptor superfamily[49]. Recently, McLatchie and collegues[32] revealed that a receptor structure with seven transmembrane domains (calcitonin receptor-like receptor, CRLR) could function as either a CGRP receptor or an adrenomedullin (AM) receptor, depending on which member of a new family of single-transmembrane-domain proteins (called receptor-activity-modifying proteins, RAMPs) was expressed. Co-expression of RAMP1 and CRLR reveals a CGRP-receptor, whereas co-expression of RAMP2 or RAMP3 and CRLR forms an AM receptor[32]. Similarly, RAMPs can interact with the calcitonin (CT)-receptor gene product to induce expression of distinct amylin receptor phenotypes[7]. In human cerebral arteries there is co-expression of CRLR and all three RAMPs[44]. The present study was designed to examine the distribution of amylin, adrenomedullin, and CGRP in the cerebral circulation of the cat, using immunocytochemistry, to examine with RT-PCR the presence of CLRL and RAMPs, and to study a possible functional role using *in vitro* and *in vivo* pharmacology.

### MATERIALS AND METHODS

#### Tissue Processing

Eight cats were anesthetized and killed by an overdose of pentobarbitone (50 mg/kg, i.p.). The brain was removed from each and the trigeminal ganglion, sections of cortical pial membranes, and vessels from the circle of Willis were dissected out. The specimens were fixed overnight by immersion in an ice-cold solution of 2% formaldehyde and 0.2% picric acid buffered to pH 7.2 with 0.1 *M* phosphate buffer. They were repeatedly rinsed in Tyrode solution containing 10% sucrose. For immunocytochemistry the specimens were frozen on dry ice and sectioned in a cryostat at 10 µm. Some of the vessels and cortical pial membranes were immersed in fixative as above, rinsed in a Tyrode solution containing 10% sucrose for 48 hr, briefly rinsed in a phosphate buffer, and stretched on chrome-alum subbed slides as whole mounts.

#### Immunocytochemistry

Cryostat sections and whole mounts were processed for the immunocytochemical demonstration of adrenomedullin[39] (B-GP 670-1), amylin[37,38] (B 29-1), and CGRP[36] (B-GP 640-1). All antibodies were used in a 1:1280 dilution and obtained from Euro-Diagnostica, Malmö, Sweden, and validated in previous studies[36,37,38,39]. The sections were exposed to the peptide antiserum for 24 hr in a moist chamber. The antigen-antibody reaction was revealed by application of fluorescein isothiocyanate (FITC)-labelled antibodies against immunoglobulin G (Dakopatts, Copenhagen, Denmark) at a dilution of 1:320 for 1 hr at room temperature. Control sections were exposed to primary antiserum that had been pre-absorbed with an excess amount of antigen (10 mg of synthetic peptide per milliliter of diluted antiserum). In addition, controls were run in which only primary antisera were used. Coexistence of peptides in nerve fibers and in ganglia was studied by a simultaneous double immunostaining procedure. In this procedure two primary antibodies were raised in different species and the secondary antibodies labelled with different fluorophores. In the present study FITC and tetramethyl rhodamine isothiocyanate (TRITC) were used. Briefly, the sections were incubated first with one peptide antiserum raised in guinea pig and with FITC-labelled IgG antibodies. In the next step the sections were incubated with a peptide antiserum raised in rabbit and then with TRITC-labelled antibodies (DAKO, Copenhagen Denmark) in a dilution of 1:80. The slides were examined and photographed in a fluorescence microscope fitted with appropriate filters for viewing FITC and TRITC fluorescence alternately.

#### Molecular Biology

The isolation of mRNA and reverse transcriptase-polymerase chain reaction assay for CRLR and RAMPs was performed using the primers and methodology as previously described[44]. As a positive control we used human right atrium. Messenger RNA was isolated from the cat MCA using the TRIzol reagent (Gibco, BRL) following the supplies instructions.

RT-PCR was carried out using the GeneAMP PCR kit (Perkin-Elmer, USA) on a DNA Thermal Cycler (Perkin-Elmer). Reverse transcription of total RNA and subsequent PCR amplification were done using the GeneAMP RNA PCR kit (Perkin-Elmer). First strand cDNA was synthesized from 1 µg total RNA in a 20-µl reaction volume using random hexamers as primers. The PCR was carried out with the following profile: 5 min at 95°C for 1 cycle, followed by 35 cycles of 30 sec at 95°C, 30 sec at 60°C, and 30 sec at 72°C; final extension was done for 7 min at 72°C. The PCR products were separated on a 1.5% agarose gel and photographed. For further details see Reference 44.

RAMP1 was also studied with Real-Time PCR. Primer Express (Applied Biosystems, Perkin-Elmer) was used to design primers to recognize human RAMP1 mRNA sequences. The nucleic acid sequence of the forward primer was CCT GGC CCA TCA CCT CTT C, and of the reverse primer CCG TAG TTA GCC TCC TGG CA.

The primers were chosen to form minimal internal structure such as primer-dimer and/or hairpin structures. Tm of the forward and reverse primers were 59° and 58.9°C, respectively, and the length of the resulting cDNA amplicon was 51 bp.

A 50-µl reaction mixture for each well was used for the Real-Time PCR (GeneAMp 5a700, PE Biosystems, Perkin Elmer, USA). The composition of the final solution was as follows: $MgC1_2$, 3 m*M*; Syber green PCR-buffer, 1X, dNTP (dATP+dCTP+dGTP+dTTP) mixture, 0.2 m*M*; Amplitaq gold, 0.025 U/µl; forward primer of RAMP1, 0.9 µ*M*; reverse primer of RAMP1, 0.9 µ*M*; and 6 µl cDNA with arbitrary concentrations of 1 (no dilution), 0.4 (2.5 times diluted), and 0.08 (12.5 times diluted).

### *In Vitro* Pharmacology

The middle cerebral artery (MCA) was removed from adult cats (n = 6) of either sex weighing between 3 and 5 kg sacrificed by exsanguination under pentobarbitone anesthesia. Ring segments, 2 mm long and 300–400 µm in diameter with intact endothelium were carefully prepared and suspended between two L-shaped metal prongs in tissue baths containing 2.5 ml of the buffer solution which was continuously gassed with 5% $CO_2$ in $O_2$ resulting in a pH of 7.4. The solutions were kept at 37°C. Isometric circular contractions were recorded through FT 03 C Grass transducers and recorded using a MacLab unit with the Chart® software. The vessels were given a passive load of 2 mN and allowed to stabilize at this level of tension for 1 ½ hr. The contractile capacities of the preparations were first tested by exposure to a buffer solution containing 60 m*M* potassium. This resulted in marked contractions 8.3 ± 1.4 mN. After two reproducible 60 m*M* potassium contractions had been obtained, prostaglandin $F_{2\alpha}$ ($10^{-6}$ *M*) was applied. This produced a steady level of contraction during which the peptides under study were applied. In parallel experiments the ability to relax to acetylcholine ($10^{-8}$ – $10^{-5}$ *M*) were taken as evidence for intact functional endothelium (data not shown). In separate tests the endothelium was removed by 10 sec of intraluminal perfusion with Triton X100[19] to study the role of the endothelium for relaxation by the peptides.

In order to examine if $CGRP_{8-37}$ acted as antagonist it was given 15 min before agonist application. During all tests 4 or 8 ring segments were run in parallel tissue baths; in blockade experiments they were exposed to the antagonist or solvent only. As analyzed in separate experiments the responses were seen to remain stable throughout the test period. The influence of prostaglandin synthesis and nitric oxide on the responses to the CGRP family of peptides using indometacin ($10^{-6}$ *M*) and L-NMMA ($10^{-6}$ *M*) treatment respectively, were used.

The data are expressed as $pEC_{50}$ which is the concentration of agonist eliciting half maximum relaxation. The differences between the values were tested for statistical significance using Student's t-test. $p < 0.05$ is considered statistical significant. All data are presented as the mean + S.D. The dissociation constant, $pK_B$, was calculated according to Tallarida et al[47].

### Solutions

The standard buffer solution used was of the following composition (m*M*): NaCl 119, KCl 4.6, CaCl 1.5, $MgCl_2$ 1.2, $NaHCO_3$ 15, Na $H_2$ $PO_4$ 1.2, and glucose 5.5. The elevated potassium buffer was obtained by a partial (50%) equimolar substitution of NaCl for KCl, resulting in a potassium concentration of 60 m*M*.

### Drugs

The following agents were used in the experiments: Human α-calcitonin gene-related peptide (hCGRP); $hCGRP_{8-37}$; amylin; adrenomedullin (all obtained from Auspep, Australia); acetylcholine, L-NMMA, indometacin, prostaglandin $F_{2\alpha}$ (Sigma, USA). All drugs were dissolved and further diluted in 0.9% saline. The concentrations are expressed as the final molar concentration in the tissue bath.

### Physiological Studies

All studies reported were carried out under a project license issued by the UK Home Office under the Animals (Scientific Procedures) Act of 1986. Eight cats (3.8 ± 0.3 kg, mean ± SD) were anesthetized with α-chloralose (60 mg/kg, i.p.) after halothane induction and ventilated with a mixture of 60% air-40% $O_2$ and placed in a stereotaxic device (David Kopf Instruments, Tujunja, CA). End-expiratory $CO_2$ was continuously monitored and adjusted by altering the stroke volume of a ventilator (Hugo-Basille); the fractional concentration of $O_2$ in inspired gas ($FIO_2$) was continuously observed (DATEX Instruments, Finland). Arterial blood gases were measured during the study. Polyethylene catheters were placed in the femoral artery and vein bilaterally for measurement of blood pressure (CWE Instruments) and administration of drugs or fluids, respectively. The level of anesthesia was monitored by observing cardiovascular responses to nociceptive stimuli and supplemental doses (15–20 mg/kg a-chloralose, i.v.) administered at two hourly intervals.

### Blood Flow Measurements

Cerebral cortical perfusion was measured continuously using laser Doppler flowmetry (LDF). The principles of operation of LDF have been thoroughly described[46] and its use in this laboratory has been described in detail[16]. Briefly, infrared light from a laser diode with a wavelength of 780 mm is directed to the surface of the brain via a fiber optic cable. The cable contains two further fibers that detect backscatter from a tissue area of approximately 1 $mm^3$. Analysis of the Doppler shift caused by that portion of light scattered by the red blood cells gives the velocity of flow for those cells, while the proportion of light backscattered is directly related to the volume of moving red cells in the brain sampled. A flow index can thus be derived from these signals. In these experiments the Moor Instruments (UK) device was used with a probe mounted in a stereotaxic manipulator (David Kopf Instruments, CA), and the signals for flow and volume continuously monitored.

Biparietal craniotomies were carried out with a low speed dental drill that was cooled with saline to prevent underlying thermal injury and the dura left intact under the probe although it was cut adjacent for drug administration. The probe was positioned over the superior aspect of the posterior parietal cortex to ensure adequate flow responses. Cerebrovascular reactivity to a brief period of hypercapnia (6–8%) was tested before collecting data and during the experiment as part of the protocol. Solutions of vehicle, amylin or adrenomedullin, were injected directly into the cortex in a volume of 1 µl using a pressure-driven glass micropipette. Doses of $10^{-7}$ *M*, $10^{-8}$ *M*, or $10^{-9}$ *M* of each of amylin or adrenomedullin, or CGRP ($10^{-8}$ *M*) were injected into separate cortical areas. Preparation of the cortex bilaterally for injection effectively doubled the possible injection sites without additional use of animals.

In order to determine accurately the changes in cerebrovascular dynamics measured with the laser Doppler the physiological variables were monitored on-line by a microcomputer. The blood pressure, heart rate, end-expiratory $CO_2$, and laser Doppler volume and flow signals were passed to a signal conditioning device and then to an analog-to-digital converter (Data Translation DT2839, UK) in an 80486-based microcomputer. Signals were all continuously monitored and displayed on the computer screen using locally written software in C. All data were stored on disk for later analysis and plotting.

### Statistics

In each animal, control data were collected prior to drug treatment and re-test. The $CBF_{LDF}$ data were converted to percentage change from the previous baseline level for the calculations[26]. The data were analyzed as a complete cohort using a single way analysis of variance (SPSS version 8)—the Dunnett's test for comparison to the vehicle injection and Scheffe's test for comparison with ha-CGRP. Significance was assessed at the $p < 0.05$ level.

## RESULTS

### Immunocytochemistry

In the trigeminal ganglion numerous nerve cell bodies of varying size contained CGRP immunoreactivity (about 40 to 50% of all neurons). Occasional amylin-ir nerve cell bodies (Fig. 1) were seen. Double immunostaining revealed that some of these co-localised with CGRP-ir while we saw no adrenomedullin-ir at all. A network of CGRP-ir nerve fibers was evident around cerebral blood vessels. Only few nerve fibers that contained amylin-ir could be visualized around the vessels (Fig. 2) while nerve fibers containing adrenomedullin-ir were not seen.

### Molecular Biology

The expression of CRLR, RAMP1, RAMP2, and RAMP3 was examined in MCA by RT-PCR. As positive control, expression of CRLR and RAMPs was found to be present in the human atrium (Fig. 3). In the cat MCA, strong signals to CRLR and RAMP3 were seen, while only traces could be detected upon UV examination of the agarose gel of RAMP1 and RAMP2. Consequently, we analysed RAMP1 with Real-Time PCR (Fig. 4). The primer pairs designed for recognition of the human RAMP1 appeared to recognize the mRNA from cat MCA with Real-Time PCR. Comparison of the peaks of the dissociation plot for the cat MCA with the human atrium, which was used as a reference, showed no difference in Tm for the two tissues, indicating no major differences in nuclei acid sequence of the amplicons.

The $C_t$-value (cut-off value) for the cat was 32.5 at a threshold of 0.5, which was much higher than the $C_t$-value of 23.2 (threshold 0.5) for the human atrial tissue at a comparable concentration (0.4), indicating a very low concen-

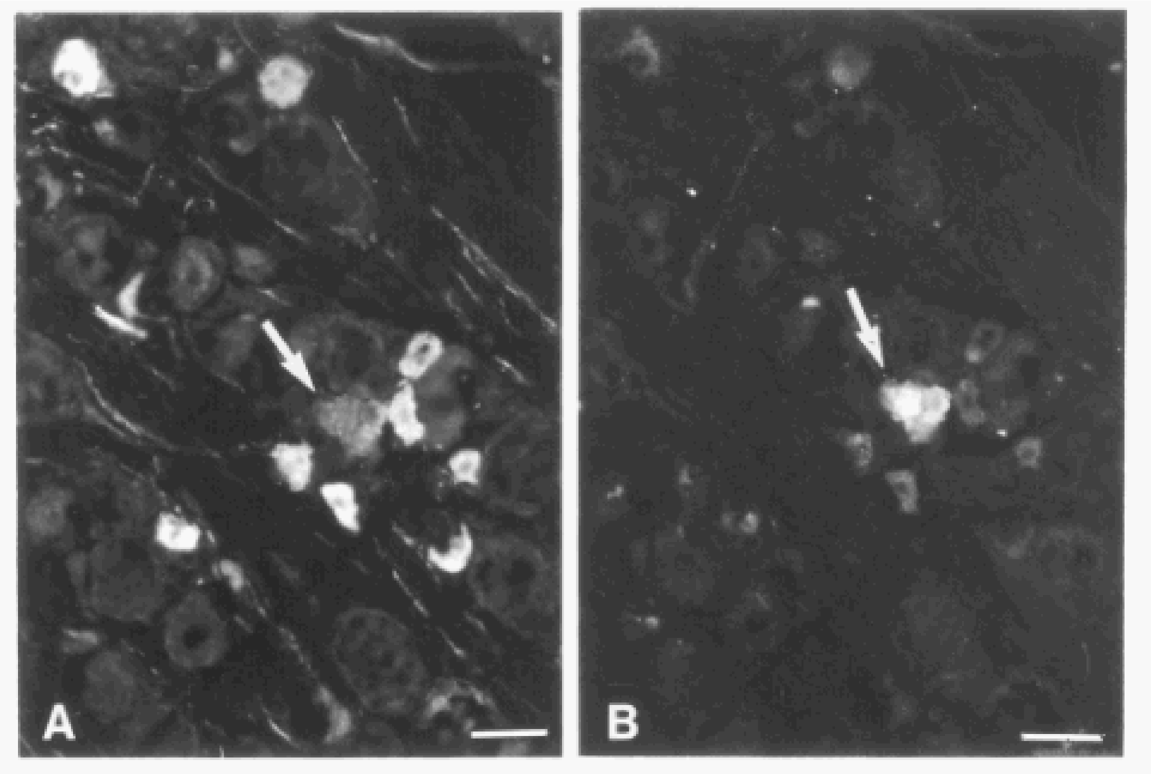

**FIGURE 1.** Cryostat section through the trigeminal ganglion of the cat showing CGRP-immunoreactivity in scattered nerve cell bodies (A). The same section processed for amylin-immunoreactivity (B) with the double immunostaining method (see Methods). Note identical distribution of cell bodies containing CGRP- and amylin-immunoreactivity (arrows).

tration of RAMP1 mRNA in cat MCA. A comparable $C_t$-value of the human atrial tissue was seen at a relative concentration of 0.00064 ($C_t$ = 32.2), i.e., at a concentration approximately 1000 times lower. However, these $C_t$-values are outside the linear range of the standard curve and can only be used as a very rough guideline.

### *In Vitro* Pharmacology

CGRP, amylin, and adrenomedullin induced relaxation of the ring segments of the cat middle cerebral artery. However, there were marked differences in reactivity to the peptides (Fig. 5). The cat MCA relaxed by 85 ± 5% (n = 10) and had a $pIC_{50}$ value of 8.3 ± 0.2 upon CGRP administration. This response was shifted toward higher a-CGRP concentrations in the presence of the antagonist ha-$CGRP_{8-37}$ ($10^{-6}$ *M*) without any significant change in maximum effect. Amylin relaxed the MCA in a concentration-dependent manner within the same dose range but with less effect (Fig. 5). Also, the amylin response was antagonized by ha-$CGRP_{8-37}$ ($10^{-6}$ *M*) in the same manner. Calculation of the $pK_B$ for $CGRP_{8-37}$ using only one concentration ($10^{-6}$ *M*) resulted in a value of 7.0 ± 0.2 and 6.9 ± 0.3 with CGRP and amylin as agonists, respectively. Only in the highest dose tested ($3 \times 10^{-7}$ *M*) was a weak relaxation seen by adrenomedullin (Fig. 5). Removal of the endothelium *in vitro* or treatment with indometacin ($10^{-6}$ *M*) or L-NMMA ($10^{-6}$ *M*) did not alter the vasodilator responses to CGRP, adrenomedullin, or amylin (data not shown).

### Cortical Blood Flow

The cardiovascular and blood gas data for the animals were within the normal range for the anesthetized cat. The respective core physiological data (n = 8, mean ± SD) were as follows: pH 7.3 ± 0.03, $pCO_2$ 4.74 ± 0.52 kPa, blood pressure 97 ± 8 mmHg. All animals reported had intact hypercapnic vasodilatation prior to data collection. Administration

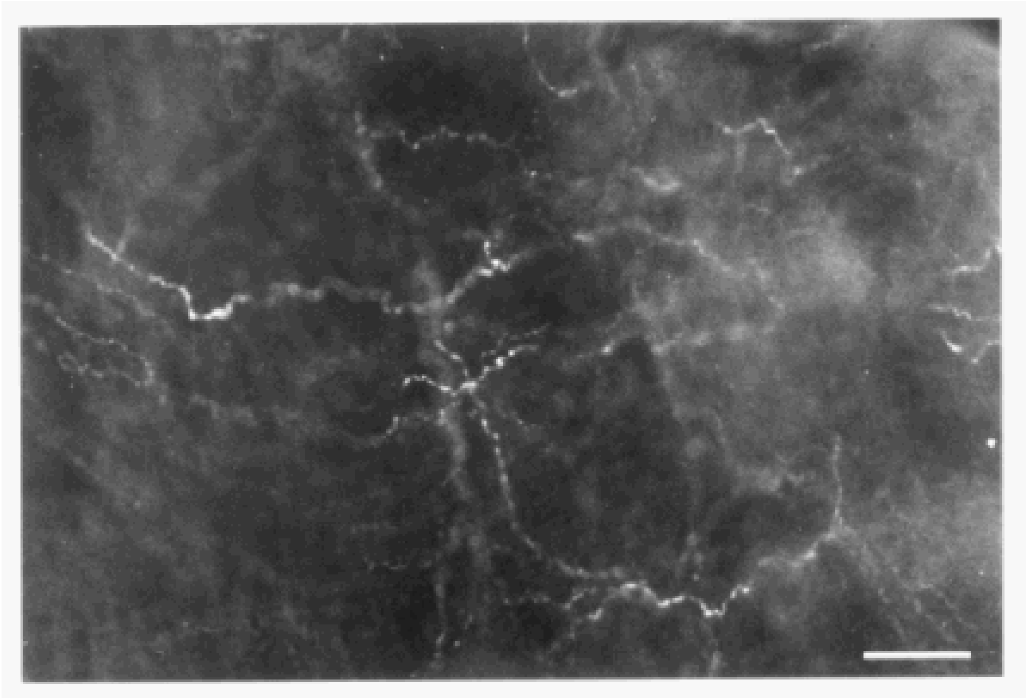

**FIGURE 2.** Perivascular amylin-immunoreactive nerve fibres in the cat. Whole mount of pial artery (×200).

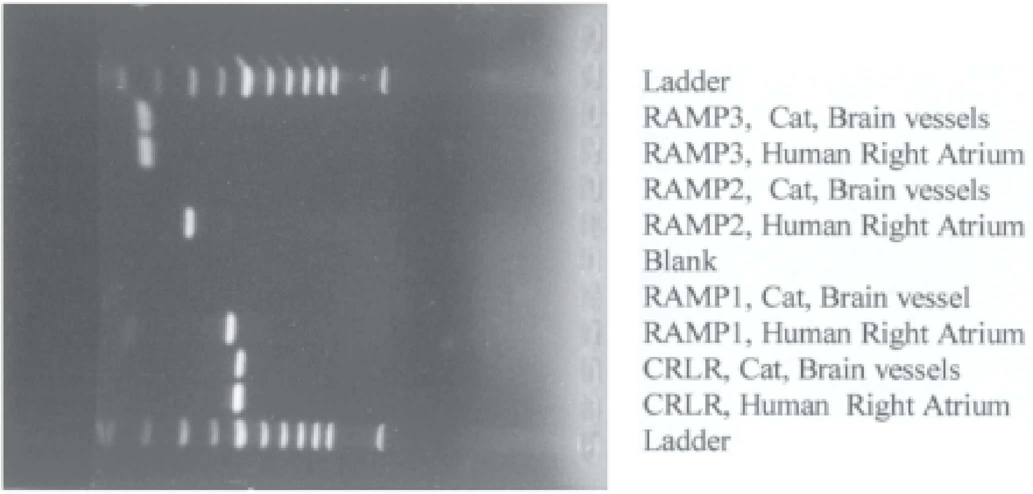

**FIGURE 3.** Expression of CRLR and RAMP1, -2, and -3 in cat MCA and human atrium demonstrated by RT-PCR. Ladder shows 100 base-pair steps. Bands corresponding to the presence of mRNA encoding CRLR as well as RAMP1, -2, and -3 (length of products were 497, 445, 283, and 159 bp, respectively) are evident for both preparations. No bands are seen in the negative control (Blank) where mRNA was not reverse transcribed to cDNA prior to amplification (lack of RT enzyme).

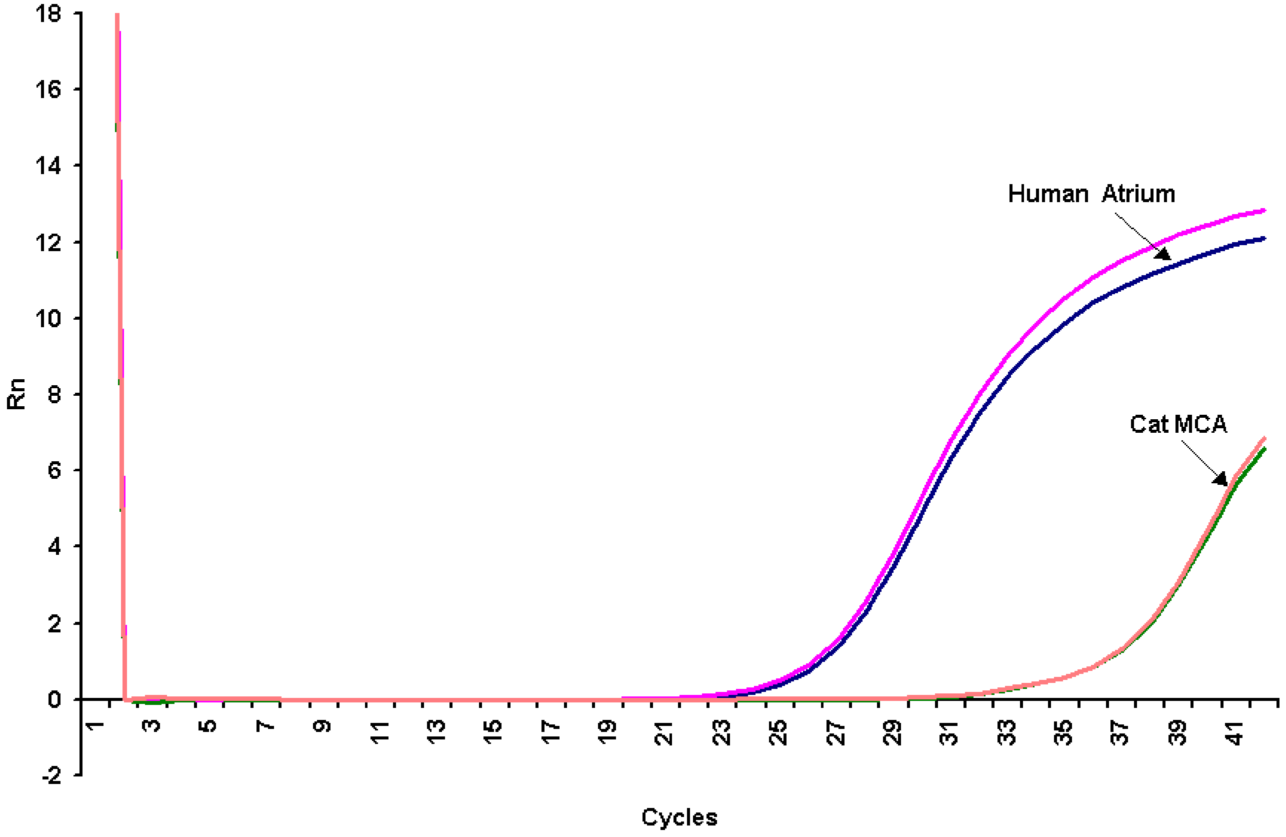

**FIGURE 4.** Real time PCR of RAMP1 in human atrial tissue (left) and cat MCA (right). Amplification plots demonstrating the number of PCR cycles (below) necessary to give an amplification that reached the cut off level ($C_t$) as detected with the ABI PRISM 5700 Sequence Detector. For each dilution, the deltaRn (the ratio for the amount of reporter dye emission to the quencing dye emission) is plotted against the cycle number. For more details see text.

locally of $CGRP_{8\text{-}37}$ did not alter the cerebral vasodilator response to hypercapnia. At the resting state CGRP ($10^{-8}$ *M*) increased the $CBF_{LDF}$ by 42 ± 6% and the vasodilatation was substantially blocked by $CGRP_{8\text{-}37}$.

Administration of the vehicle in the amylin series of cortices demonstrated a change of 7 ± 1 % (n = 5) for $CBF_{LDF}$ (Fig. 6). The maximum response seen with local injection of amylin ($10^{-7}$ *M*) was a 32 ± 4% (n = 5) increase in $CBF_{LDF}$. The responses to amylin were significant as a cohort ($F_{5,26}$ = 24, $p < 0.05$). In turn, both doses of $10^{-7}$ *M* and $10^{-8}$ *M* ($p < 0.05$, Dunnett's test) were significantly different from vehicle while $10^{-9}$ *M* ($p = 0.11$, NS) was not (Table 1).

The dose of $10^{-7}$ *M* was re-tested to produce a 30 ± 3% (n = 5) change which was reduced to 19 ± 2% after local administration of $CGRP_{8\text{-}37}$ $10^{-6}$ *M* ($p < 0.05$).

Administration of vehicle in the adrenomedullin series of cortices demonstrated a change on 7 ± 2% (n = 5) for $CBF_{LDF}$. The mean maximum response seen with local injection of adrenomedullin ($10^{-7}$ *M*) was 15 ± 2% (n = 5; $p < 0.05$). No effect significant from vehicle was seen with adrenomedullin $10^{-8}$ or $10^{-9}$ *M* (n = 5, respectively). After administration of $CGRP_{8\text{-}37}$ $10^{-6}$ *M* the response to adrenomedullin $10^{-7}$ *M* was 14 ± 2% (n = 5), which was not different to the pretreatment level.

### DISCUSSION

Amylin, CGRP, and adrenomedullin belong to a family of structurally related peptides. They have in common a six or seven amino acid ring structure linked by a disulfide bridge as well as being C-terminally amidated. The C-terminal part of adrenomedullin exhibits approximately 25% homology with CGRP and amylin. Amylin mRNA has been demonstrated in extracts from rat dorsal root ganglia[41]. Recently, using *in situ* hybridization and immunocytochemistry,

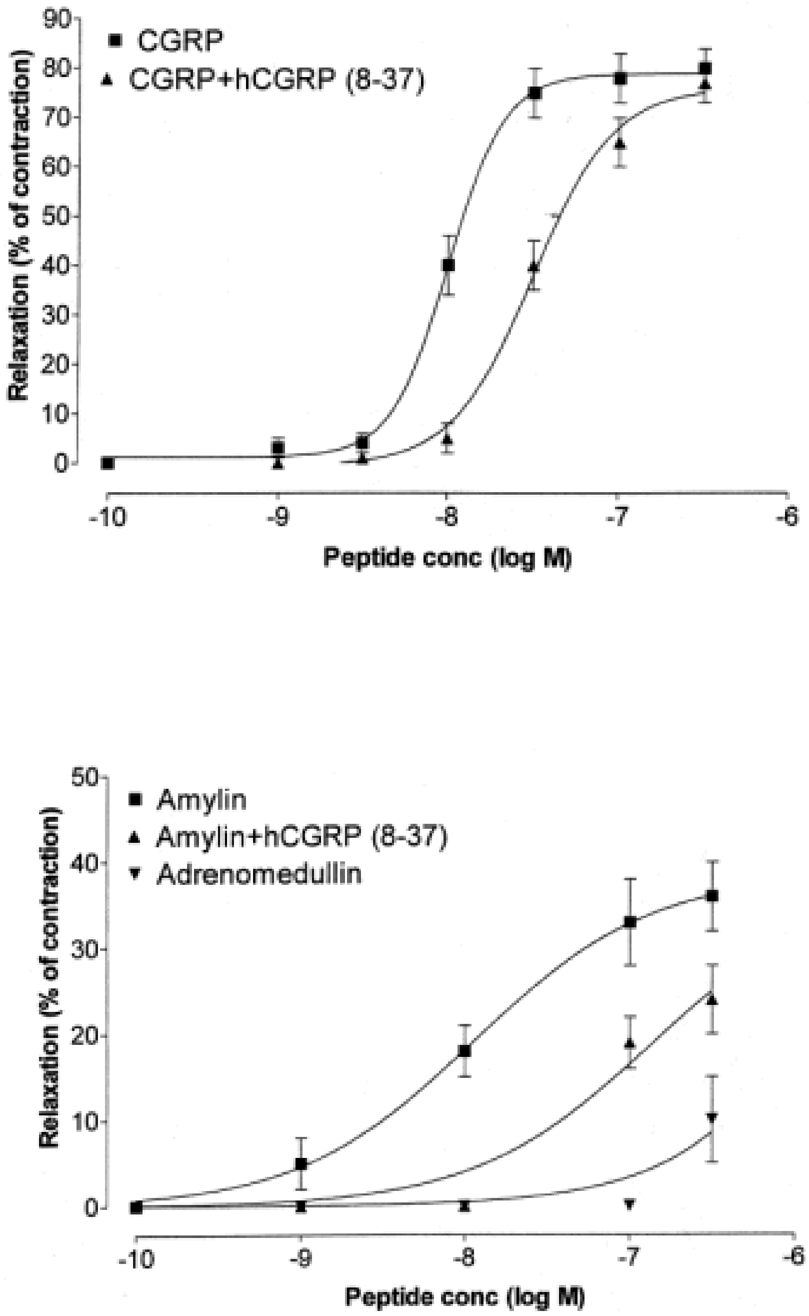

**FIGURE 5.** Relaxant response in cat middle cerebral arteries precontracted by 3 μ*M* prostaglandin $F_{2\alpha}$ to the cumulative application of (A) aCGRP in the presence or absence of 1 μm $aCGRP_{8\text{-}37}$, and (B) amylin (with or without $aCGRP_{8\text{-}37}$) and adrenomedullin. Values given represent mean + S.D., n = 6.

amylin was found in a population of small to medium sized nerve cell bodies in sensory ganglia in the rat[36]. In the present study, we observed in the cat a subpopulation of amylin-ir nerve cell bodies in the trigeminal ganglion. Also it was seen that the amylin-containing nerve cell bodies constituted a subpopulation of those expressing CGRP. Amylin-ir containing nerve fibers have been seen in the dorsal horns of the spinal cord and, to a lesser extent, in peripheral tissues receiving sensory innervation[36]. We found amylin-ir fibers in cerebral vessels, and it is reasonable to

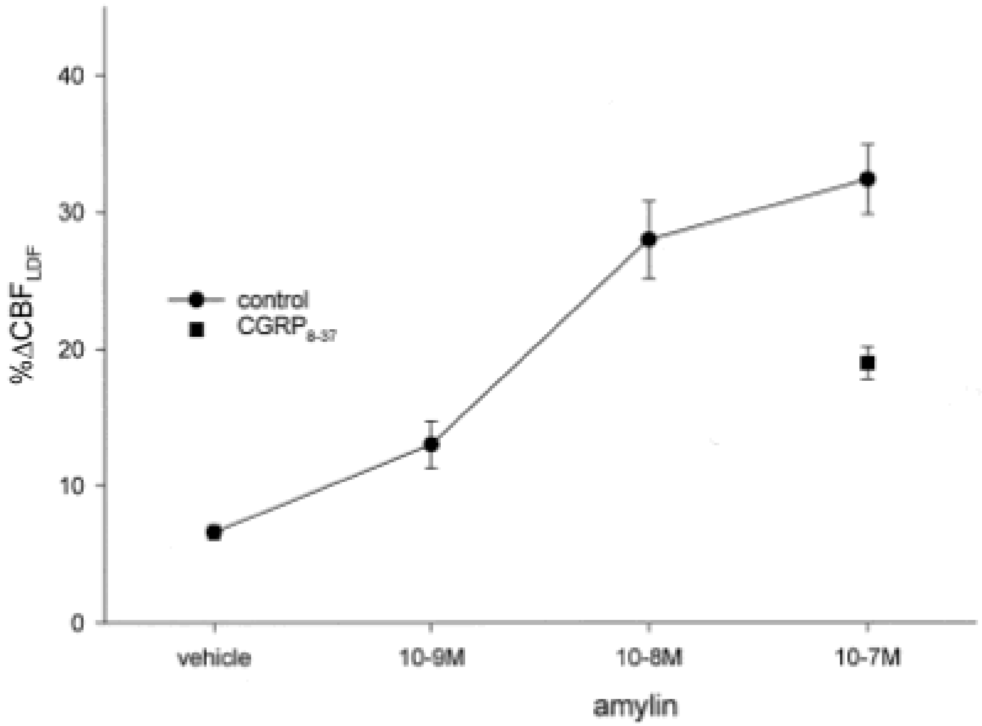

**FIGURE 6.** Response of local cortical blood flow to injection of amylin. There is a concentration dependent increase in cortical blood flow measured by laser Doppler flowmetry ($CBF_{LDF}$) with a maximum after amylin $10^{-7}$ *M*. This effect is antagonised by the CGRP receptor blocker $CGRP_{8-37}$.

**TABLE 1**
**Effect of Local Injection of Amylin or Adrenomedullin on Cortical Blood Flow**

| Peptide | Concentration (*M*) | $\%\Delta CBF_{LDF}$ (mean ± SEM) |
|---|---|---|
| Amylin | $10^{-7}$ | 32 ± 3 |
| | $10^{-8}$ | 28 ± 3 |
| | $10^{-9}$ | 13 ± 2 |
| Adrenomedullin | $10^{-7}$ | 15 ± 2 |
| | $10^{-8}$ | 10 ± 2 |
| | $10^{-9}$ | 9 ± 2 |

presume that they originate in the trigeminal ganglion, since studies of other cranial ganglia have been negative (unpublished data).

The molecular biology experiments demonstrated the presence of mRNA for CLRL and all three RAMPs in the cat MCA; however, RAMP1 and RAMP2 showed low signals. This might be due to a difference between feline and human RAMPs or low levels of these RAMPs in the feline tissue. With Real-Time PCR we observed the presence of RAMP1 in feline MCA but in comparison with the human atrium the level was low. The latter observation agrees well with data obtained from human brain vessels[44]. Furthermore, there are studies that have demonstrated the expression of CGRP[8] and adrenomedullin receptors mRNA[33] in the cerebral circulation. However, the next question we addressed was if the mRNA is translated into functional receptors.

Amylin, adrenomedullin, and CGRP exert their effects by increasing the tissue level of cAMP[35]. It has been suggested that most of the reported effects of amylin can be explained through its interaction with CGRP receptors[27]. There are some indications that the CGRP blocker $CGRP_{8-37}$ can antagonize the responses to amylin, adrenomedullin, and CGRP[43]. In rat cerebral arterioles adrenomedullin has been shown to induce a concentration-dependent vasodilation, probably acting through CGRP receptors since this dilation was supressed by pretreatment with the CGRP receptor antagonist $CGRP_{8-37}$. In contrast, amylin did not cause vasodilation at concentrations up to $10^{-6}$ *M*[34].

In the cat MCA we observed that amylin, adrenomedullin, and CGRP caused relaxation in the presence or absence of the endothelium. The relaxations induced by amylin and CGRP were not significantly attenuated by L-NMMA or by indomethacin. This is in agreement with previous studies of cerebral vessels for CGRP where the relaxation has been shown to occur in parallel with activation of adenylyl cyclase[9,10]. In other vascular regions the situation may differ. Inhibition of nitric oxide synthase reduced the adrenomedullin-induced hypotension and the hindquarter perfusion pressure of the rat[12]. Furthermore, the relaxant effect of CGRP in the rat thoracic aorta was dependent on the presence of the endothelium[4] and could be blocked by haemoglobin, methylene blue[13,] or L-arginine analogues[17,20], but not by indomethacin[13]. The results suggest that adrenomedullin and CGRP have similar inhibitory effects in rat aorta and that the effects are mediated by nitric oxide. On the other hand, the responses to amylin and CGRP in cat cerebral arteries is independent of the endothelium[10]. This agrees well with studies of adrenomedullin and CGRP in the porcine coronary artery[45,48]. Previously, we have shown that CGRP relaxes cerebral vessels via the accumulation of cAMP[10]. This is in concert with two reports on pig coronary artery demonstrating that adrenomedullin and CGRP decrease intracellular $Ca^{2+}$ and $Ca^{2+}$ sensitivity[14,30].

We observed that amylin and CGRP caused a concentration-dependent increase in CBF, and that the effect was blocked by $CGRP_{8-37}$. The maximum responses to amylin and CGRP were 32 ± 4% and 42 ± 6%, respectively, while adrenomedullin only increased the blood flow by 15 ± 2% (vehicle caused 7 ± 2%). $CGRP_{8-37}$ had no effect on the adrenomedullin response. The lack of effect of $CGRP_{8-37}$ on the adrenomedullin response agrees well with that seen in other vascular beds[15,21] and in cultured human endothelial cells[28], suggesting the existence of a population of adrenomedullin receptors that are distinct from the CGRP receptors[43].

There are few previous studies examining the effects of amylin and adrenomedullin on CBF. Baskaya et al.[2] performed a comprehensive study in the dog showing similar potency of adrenomedullin and CGRP while amylin was 100 times less potent. This stands in contrast to our findings in the cat which had the following potency order, CGRP = amylin > adrenomedullin. Clearly, this suggests species differences in the CGRP receptor profile for these peptides. *In vivo* studies[2] have shown that NOS inhibition is ineffective, while cAMP increases and that pretreatment with $CGRP_{8-37}$ abolishes the adrenomedullin response. This is in agreement with our studies of CGRP *in vitro*[10,24]. The lack of effect of $CGRP_{8-37}$ on adrenomedullin responses in the cat (present data) as compared to the dog[2] may be due to a difference within the CGRP receptor population. Adrenomedullin had only weak dilator effects in our study. Since the amylin response was blocked by $CGRP_{8-37}$ it further indicates that the cerebrovascular effects that have been observed are likely to be mediated via activation of CGRP receptors. This issue will not be resolved until specific agonist or antagonist compounds have been developed.

In the present study, we found that $CGRP_{8-37}$ antagonized the relaxant effects of amylin and CGRP both *in vitro* and *in vivo*, but not adrenomedullin *in vivo*. The responses to adrenomedullin were very weak and seen only at the highest concentrations tested. $CGRP_{8-37}$ is a competitive antagonist of CGRP-induced relaxant responses, providing a $pK_B$ value of around 7.0. This agrees well with the present observations. Amylin also relaxed the cat middle cerebral artery in a concentration-dependent manner and $CGRP_{8-37}$ caused a comparable $pK_B$ value. Thus, it is likely that CGRP and amylin act at the same CGRP receptor in cat cerebral arteries.

In the porcine coronary artery adrenomedullin caused relaxation, but this was not antagonized by $CGRP_{8-37}$[48]. However, in isolated rat heart the adrenomedullin-induced vasodilation was blocked by $CGRP_{8-7}$ suggesting activation of CGRP receptors[11]. In human vascular endothelial cells adrenomedullin increases the accumulation of cAMP and this was antagonized by $CGRP_{8-37}$[28]. The weak relaxant effect of adrenomedullin in cat middle cerebral arteries is supported by a recent study in which adrenomedullin receptor mRNA could not be detected in human brain vessels[33]. However, in spite of the lack of expression of adrenomedullin receptor mRNA, the peptide exerted activation of adenylyl cyclase, probably through activation of CGRP receptors[33]. Thus, the parallelism with the present data suggests the presence of CGRP receptors in cat MCA, and that both amylin and adrenomedullin may be able to interact with the CGRP receptors to induce the vascular responses.

In conclusion, the study has documented the presence of amylin-ir in the cerebral circulation and in a subset of cells in the trigeminal ganglion where it is co-localised with CGRP-ir. In isolated cerebral arteries amylin caused dilatation and *in vivo* amylin increased cerebral flow, each indicating a functional effect for amylin. The response seems, however to be mediated via the CGRP receptor since the available antagonist had the same effect on both amylin and CGRP responses. Further dissection of any role for amylin will require better selective agonists and antagonists to allow the complex relationships to be disentangled. Certainly, amylin containing trigeminal neurons must be included in any complete formulation of the physiology of the neural innervation of the cerebral circulation.

### ACKNOWLEDGEMENTS

This work was supported by the Swedish Medical Research Council (grant no. 05958) and the Welcome Trust and the Migraine Trust. PJG is a Wellcome Trust Senior Research Fellow.

| | | |
|---|---|---|
| Received: | January | 5, 2001 |
| Accepted: | March | 19, 2001 |
| Published: | May | 1, 2001 |
